# Quantitative but Not Qualitative Differences: A Longitudinal Analysis of Grammatical Marker Development in Mandarin‐Speaking Autistic Children

**DOI:** 10.1002/aur.70195

**Published:** 2026-02-07

**Authors:** Ziyan Meng, Li Wang, Hoyee W. Hirai, Patrick C. M. Wong

**Affiliations:** ^1^ Department of Linguistics and Modern Languages The Chinese University of Hong Kong Hong Kong SAR China; ^2^ Department of Educational Psychology The Chinese University of Hong Kong Hong Kong SAR China; ^3^ Brain and Mind Institute, The Chinese University of Hong Kong Hong Kong SAR China

**Keywords:** autism, language development, longitudinal, Mandarin, morphosyntax

## Abstract

Past research has revealed large differences between typically developing (TD) and autistic children's language development. However, little is known about whether such differences are quantitative or qualitative, especially in the morphosyntactic domain. This study is the first longitudinal research aiming to systematically investigate the developmental patterns of a wide range of Mandarin grammatical markers in autistic children. The mastery of target markers in autistic children (*N* = 88, M_age_ = 44.9 m, Range = 26–76 m) was assessed longitudinally across three time points using parent reports and compared with that of TD children (*N* = 84, M_age_ = 23.2 m, Range = 16–30 m) assessed at a single time point. We further examined the influence of autism severity and initial language ability. The results suggested that autistic children acquired Mandarin grammatical markers in a typical sequence but at a slower rate. Additionally, this developmental pattern was maintained regardless of autism severity and initial language ability. These findings suggest that autistic children's language development differs quantitatively but not qualitatively from that of TD children, reflecting developmental delay rather than deviance.

## Lay Summary

In this study, we found more similarities than differences in terms of language development of autistic children relative to typically developing children. Specifically, we found that autistic children acquired Mandarin grammatical markers in a sequence similar to that of TD children. Even those with more pronounced autism symptoms or lower initial language ability still followed this typical acquisition order. Furthermore, though there was a delay, our results suggested that autistic children might eventually catch up and acquire most of the grammatical markers.

## 1 Introduction

Though language difficulty is not a necessity for a diagnosis of autism (American Psychiatric Association 2013), it is one of parents' earliest concerns when seeking a diagnosis and intervention for their child (Kim et al. 2014). Understanding the nature of these language difficulties—specifically, whether they represent a quantitative delay or a qualitative difference—is critical for supporting clinical decisions and designing effective interventions. A quantitative delay is defined as acquiring skills in a typical developmental sequence but at a slower pace (Hare‐Harris et al. 2019). By contrast, a qualitative difference is characterized by nonsequential skill acquisition, which is also described as developmental deviance (Hare‐Harris et al. 2019). In previous autism research on language development, the debate between quantitative and qualitative difference hypotheses remains unresolved, with varying degrees of empirical support (e.g., Ellis Weismer et al. 2011; Su and Naigles 2023; Zhou, Ma, and Zhan 2019; Zhou, Zhan, and Ma 2019; Ge et al. 2023).

Due to their functional complexity, grammatical markers are generally challenging to acquire in early language development (Huang et al. 2022), making them a particularly informative linguistic domain for investigating the quantity versus quality distinction in autism. In typically developing (TD) children, the acquisition of grammatical markers follows a highly systematic and predictable developmental sequence, which has been well documented across languages, including English (Brown 1973; de Villiers and Villiers 1973), Spanish (Miguel 1989), Italian (Caprin and Guasti 2009), and Mandarin (Huang et al. 2022). This robust cross‐linguistic regularity provides a strong developmental benchmark: delayed mastery while preserving the canonical sequence would be consistent with a quantitative delay, while deviations from the established acquisition order would suggest qualitative differences.

This study focuses on Mandarin Chinese. Unlike English, Mandarin employs a distinct grammatical marker system characterized by the absence of inflectional morphemes. The typical developmental sequence of Mandarin markers has been well documented in Huang et al. (2022). Based on data from 338 Mandarin‐speaking TD children aged 17 to 36 months, they identified a clear acquisition order: negative *mei*, *bu*, and possessive *de* were acquired the earliest, while aspect *zheng* was acquired the latest. Their results provide a clear baseline for comparing the developmental patterns in Mandarin‐speaking autistic children.

Previous studies on grammatical marker acquisition in autistic children have yielded mixed findings. Bartolucci et al. (1980) and Howlin (1984) investigated autistic children's developmental pattern of English grammatical markers using small datasets, with sample sizes of 10 (M_age_ = 10.9 years) and 16 (M_age_ = 7.9 years), respectively. Both studies found that the acquisition sequences observed in autistic children were distinct from those of TD children, indicating qualitative deviations across groups. In contrast, Park et al. (2012), also using a small but younger sample (*N* = 17, M_age_ = 56.18 m), reported a significant association between TD and autistic children's acquisition orders, which demonstrated a quantitative but not qualitative difference between groups. The discrepancy between these findings may be attributed to methodological limitations, including small sample sizes and cross‐sectional designs prone to ceiling effects. Moreover, it is also important to note that the existing findings are exclusively based on English. Given the profound differences between Mandarin and English grammatical marker systems, it remains unclear how grammatical markers develop in Mandarin‐speaking autistic children. Our study directly addresses this gap by conducting, to our knowledge, the first longitudinal investigation of grammatical marker development in a large sample of Mandarin‐speaking autistic children. This design overcomes the ceiling effects of previous studies and provides new insights into an understudied linguistic population.

Furthermore, the role of child‐based factors, such as autism severity and initial language ability, in modulating the developmental trajectories of grammatical markers is still unclear. While some studies reported that autism severity, as measured by the ADOS‐2 calibrated severity score, negatively predicted the growth of autistic children's language ability (Charman et al. 2005; Ellis Weismer and Kover 2015), others found that this relationship disappeared after controlling for nonverbal cognition (So and Song 2023; Nevill et al. 2019). Conversely, evidence on the role of children's initial language ability was more robust, which consistently suggested that autistic children's early language skills positively predicted subsequent language development (e.g., So and Song 2023; Fusaroli et al. 2019; Tek et al. 2014). A comprehensive longitudinal investigation that accounts for these two factors is necessary to clarify their influence.

In summary, most previous studies on grammatical marker development were cross‐sectional and had small sample sizes. Moreover, they paid little attention to languages other than English. To address these limitations, this study systematically investigated the developmental patterns of a wide range of grammatical markers in Mandarin‐speaking autistic children using a large longitudinal dataset. We adopted the parent reporting approach established by Huang et al. (2022). It is important to note that while parent reports are effective for tracking the emergence of language features, they may not fully capture nuanced mastery, such as the ability to distinguish imitative or formulaic language use. However, the method we employed, following Huang et al. (2022), does not directly ask parents whether their child produces specific target markers. Instead, parents are asked to select the sentence that most closely matches their child's current (rather than the best) language complexity level. Thus, this approach can, to some extent, provide insights into children's actual language abilities.

### 1.1 The Present Study

This study aimed to systematically examine the developmental patterns of a wide range of Mandarin grammatical markers in Mandarin‐speaking autistic children. Particularly, we addressed three questions:

1. What is the pattern of autistic children acquiring Mandarin grammatical markers?
2. Is this pattern the same as that of TD children?
3. How do the two factors, namely autism severity and initial language ability, affect the pattern?

Based on the findings of Park et al. (2012) with younger autistic children, we predicted that while autistic children would acquire Mandarin grammatical markers with a quantitative delay, their acquisition sequence would not qualitatively differ from that of TD children. Furthermore, the factors of autism severity and initial language ability would affect the development quantitatively (i.e., the developmental speed) but not qualitatively (i.e., the developmental sequence).

## 2 Method

### 2.1 Participants

The total sample (*N* = 172) included two groups: (a) children who had a prior clinical diagnosis of autism or were considered by clinicians to have a high likelihood of autism, with their diagnostic status subsequently confirmed using the ADOS‐2 at study entry (autism group, *N* = 88), and (b) typically developing children (TD group, *N* = 84).

The autistic participants were drawn from an ongoing longitudinal program designed to introduce caregivers to strategies for improving their autistic child's social communication outcomes (see Wang et al. 2025, 2026; Wong et al. 2025 for more details on this program). All children were Chinese‐speaking (including Mandarin and Cantonese) and were aged 24–60 months at enrollment. Their diagnosis was confirmed by our team using the Autism Diagnostic Observation Schedule, Second Edition (ADOS‐2; Lord et al. 2012). They had not yet developed three‐ or more‐word phrase speech (i.e., using ADOS‐2 Module 1 or ADOS‐2 Module 2 A1 item > 0), indicating limited spontaneous expressive language. Based on parent reports, they did not have severe hearing or visual impairment or any neurological or psychiatric conditions requiring medication. To be involved in the current study, additional inclusion criteria were applied: they were required to be Mandarin speaking and eligible to complete the Word and Sentence form of the Chinese Communicative Development Inventory Putonghua (Mandarin) version (CCDI‐P; Tardif et al. 2008). Since the Word and Sentence form is typically designed for children aged 16–30 months and presumes basic productive vocabulary, the participants in this study were assessed only after their vocabulary production percentile score reached 75% relative to the 16‐month‐old norm on the CCDI‐P Words and Gestures form. For some children who did not meet this vocabulary criterion at initial enrollment in the larger program, their first CCDI‐P assessment was administered later. This resulted in an age range of 26–76 months at Time 1 (*N* = 88, M_age_ = 44.9 m, SD = 11). These children were assessed longitudinally at three time points. Due to sample attrition, data were available from 66 children at Time 2 (M_age_ = 49.8 m, SD = 9.5), and from 32 children at Time 3 (M_age_ = 61.9 m, SD = 9.9).

Although Huang et al. (2022) have established a typical sequence for Mandarin grammatical markers, this remains, to our knowledge, the only empirical study to have done so. To rigorously evaluate grammatical marker acquisition in autism, it was therefore necessary to first verify the robustness of the reported sequence within an independent sample of TD children drawn from the same linguistic context. Accordingly, a TD group was recruited through social media platforms. To be eligible for this study, children were required to meet the age criteria (i.e., 16–30 months) for the Word and Sentence form of the CCDI‐P and have no severe hearing or visual impairments. They were also categorized as little‐to‐no concern for autism based on the ADOS‐2 Toddler Module (Lord et al. 2012). Furthermore, participants demonstrated no signs of language delay, as indicated by vocabulary production percentile scores of 25% or higher on the CCDI‐P. As a result, a total of 84 eligible Mandarin‐speaking TD children were recruited, with an average age of 23.2 months (SD = 4). TD participants were assessed at a single time point. This cross‐sectional design was sufficient for the current objective of confirming the relative acquisition order reported by Huang et al. (2022) and providing a contemporaneous control group for comparison with autistic children. This approach enabled a rigorous yet efficient validation of the typical developmental sequence while minimizing participant burden.

Table 1 presents the participants' demographic information by group. It is important to note that the TD and autism groups differed in regard to sex (the autism group included more males, *p* < 0.001), maternal education (the TD group had higher education levels, *p* < 0.01), and family income (the TD group had higher incomes, *p* < 0.001). Accordingly, these factors were included as potential demographic covariates in the analyses. Regarding language ability, the autistic children exhibited lower vocabulary production and sentence complexity scores than the TD children at Time 1 (*ps* < 0.001). However, no significant differences were observed between the two groups for these measures at Time 2 and Time 3 (*ps* > 0.05). This indicated that the selected time points effectively captured three distinct phases of language development in the autism group: initially lower than the TD group, then comparable to it, and finally slightly (though not significantly) higher.

**TABLE 1:** Participants' demographic information.

		Autism group			TD group	Autism versus TD
		Time 1	Time 2	Time 3		
*N*		88	66	32	84	
Sex	Female	14	10	8	44	*p* < 0.001
	Male	74	56	24	40	
Age	Mean (SD)	44.9 (11)	49.8 (9.5)	61.9 (9.9)	23.2 (4)	TD < Autism^a^
	Range	[26–76]	[30–65]	[43–77]	[16–30]	
CDI (Raw)	Vocabulary production (max = 799)	372 (201.5)	521.3 (192.2)	669.9 (121)	535.7 (239.9)	T1 < TD^a^
	Sentence complexity (max = 81)	30.7 (21.3)	46.9 (22.5)	61 (19.9)	49.8 (26.3)	T1 < TD^a^
ADOS CSS		6.6 (1.5)	—	—	—	
MSEL verbal IQ		68.2 (29.4)	—	—	—	
Maternal education	High School and below	5	—	—	1	TD > Autism^b^
	College and Bachelor	58	—	—	42	
	Master and above	23	—	—	41	
	Unknown	2	—	—	0	
Annual Household Income (RMB)	100,000 and below	8	—	—	5	TD > Autism^a^
	100,001–200,000	19	—	—	2	
	200,001–300,000	15	—	—	8	
	300,001–500,000	27	—	—	26	
	500,001 and above	18	—	—	43	
	Unknown	1	—	—	0	

*Note:* Values are presented as mean (SD) for continuous variables and *N* for categorical variables. Continuous variables were compared between groups using linear‐mixed effects models, and categorical variables were compared using chi‐squared tests.

Abbreviations: ADOS‐2 = Autism Diagnostic Observation Schedule, Second Edition; CCS = Calibrated Severity Score; MSEL = Mullen Scales of Early Learning; SD = standard deviation.

^a^ *p* < 0.001.

^b^ *p* < 0.01.

### 2.2 Measures

#### 2.2.1 Autism Diagnostic Observation Schedule, Second Edition (ADOS‐2; Lord et al. 2012)

The ADOS‐2 is a play‐based tool used to assess symptoms and behaviors associated with autism. It includes different modules tailored to different age ranges and language levels. After completing Module 1 to Module 3, a calibrated severity score (CSS) is obtained. The ADOS‐2 CSS ranges from 1 to 10, which corresponds to four levels of autism spectrum‐related symptoms: 1–2 denoting minimal‐to‐no evidence, 3–4 representing a low level, 5–7 indicating a moderate level, and 8–10 indicating a high level. The Toddler module does not provide a CSS. Instead, it generates an overall total score, which corresponds to three levels of concern: little‐to‐no, mild‐to‐moderate, and moderate‐to‐severe.

Among the 88 children in the autism group, 85 were assessed using the ADOS‐2 Module 1 or Module 2 by a qualified clinical psychologist with clinical and research reliability for conducting the ADOS‐2. The remaining three children could not attend in‐person assessments due to COVID‐19 restrictions. As a result, we confirmed these children's diagnoses and obtained their ADOS‐2 CSS (Table 1). For the purpose of exploring the effect of autism severity, these children were assigned to either ADOS‐H or ADOS‐L subgroups based on their ADOS‐2 CSS. More specifically, children whose ADOS‐2 CSS ranged from 8 to 10 were placed in the ADOS‐H subgroup (*N* = 23 at Time 1, 14 at Time 2, 6 at Time 3), indicating that they had a higher level of autism spectrum‐related symptoms. The remaining autistic children, whose ADOS‐2 CSS ranged from 4 to 7, were assigned to the ADOS‐L subgroup (*N* = 62 at Time 1, 51 at Time 2, 25 at Time 3). Regarding the TD group, all 84 participants were evaluated using the ADOS‐2 Toddler module, and they were all categorized as little‐to‐no concern of autism.

#### 2.2.2 Chinese Communicative Development Inventory Putonghua (Mandarin) version (CCDI‐P; Tardif et al. 2008)

The CCDI‐P is a parent report instrument measuring children's early language development. It is modeled on the original MacArthur–Bates Communicative Development Inventories (Fenson et al. 2007), and has been normed for Mandarin. Similar to the original CDI, the CCDI‐P has two forms: *Words and Gestures* (age range: 8–16 months), and *Words and Sentences* (age range: 16–30 months). In this study, all the participating autistic families and the TD control group had completed the Words and Sentences form. Two indices, namely vocabulary production and sentence complexity raw scores, were derived (Table 1).

#### 2.2.3 Mullen Scales of Early Learning (MSEL; Mullen 1995)

The MSEL is a standardized assessment measuring children's cognitive and language ability. It consists of five subscales, namely gross motor, visual reception, fine motor, receptive language, and expressive language, and each of them is standardized to calculate a T‐score. The T‐score of each subscale corresponds to five descriptive categories: very low (score range: 20–30), below average (31–39), average (40–60), above average (61–69), and very high (70–80). The sum of receptive and expressive language T‐scores constitutes a verbal IQ score.

In this study, 85 out of 88 autistic children (96.6%) completed the MSEL assessment at the recruitment stage, and three children did not attend due to COVID‐19 restrictions. Their verbal IQ scores are reported in Table 1, and were used to index their initial language ability. For the purpose of this study, we divided the autistic children into subgroups using a verbal IQ score of 60, because a T‐score of less than 30 on a subscale represents a very low level of ability (with a percentile rank of 1 and 2). Thus, the autistic children whose verbal IQ T scores were above 60 were classified as the Verbal‐H subgroup (*N* = 34 at Time 1, 30 at Time 2, 18 at Time 3). Otherwise, they were classified as the Verbal‐L subgroup (*N* = 51 at Time 1, 35 at Time 2, 13 at Time 3).

### 2.3 Grammatical Marking Rescoring (Huang et al. 2022)

In the Words and Sentences form of CCDI‐P, there is one section measuring children's grammatical complexity, where parents are asked to choose the sentence that most closely matches their child's language complexity level. There are 27 items in total, involving nine categories of target grammatical markers: negations, modals, resultative verb compounds, aspects, possessives, classifiers, adverbs, sentence final particles, and complex clauses (Table 2). Based on parents' responses, we analyzed their child's mastery of each grammatical marker using the grammatical marking rescoring method developed by Huang et al. (2022).

**TABLE 2:** Description of Mandarin grammatical markers involved in this study (Li and Thompson 1981; Huang et al. 2014, 2022).

Category	Marker	Remark	Example
Negation	Mei (没)	Denoting denial or nonexistence	Mei zou. (have not left)
	Bu (不)	Negate an attribute, or the state of an object, or an auxiliary verb	Bu neng zou. (cannot leave)
	Bie (别)	Only used in negative imperatives	Bie zou. (do not leave)
Aspect*	Le (了)	Perfective	Ta kan le na ben shu. (he read that book)
	Yao (要)	Future	Qiu yao diao. (ball will fall)
	Guo (过)	Experiential	Ta kan guo na ben shu. (he has read that book before)
	Zheng (正)	Imperfective	Wo zheng chifan ne. (I am eating)
Modal	For example, yao, xiang, neng, hui	Expressing desire, ability, etc.	Baobao xiang yao qiu. (baby want ball)
Resultative verb compound (RVC)	For example, da‐sui	Verbs consisting of two or more morphemes indicating an action and its results, direction, completion, and achievement	Ta da‐sui le huaping. (he broke the vase)
Adverb	For example, zai, hai, you	Denoting frequency	Zai chi dian. (eat some more)
Sentence final particle (SFP)	For example, ne, ma, le	Indicating the current status of an action (modality of the utterance) or indicating a question	Neng wan ma? (can play?)
Complex clause	For example, gei	Double object	Mama gei baobao jiang gushi. (mom tells baby a story)
Possessive	De (的)	Owner + de + owner's property	Mama de shu. (mom's book)
Classifier	For example, ge	Number + classifier + noun	Yi ge ren. (one person)

*Note:* *“Aspect” refers to how an event is perceived, unlike “tense,” which refers to the time of the reported event in relation to the speech time (Wang and Sun 2015).

Take the Mandarin possessive marker *de* as an example. In Item 4, parents were asked “which sentence most closely corresponds to your child's way of expressing possession?” Four options were provided to choose from: (a) 不会说 (“cannot say”), (b) 宝宝车 (“baby car”, noun + noun), (c) 宝宝的车 (“baby's car”, noun + possessive marker *de* + noun), and (d) 我的车 (“my car,” pronoun + possessive marker *de* + noun). In this case, both options (c) and (d) denoted that the child was able to produce the possessive marker *de* (i.e., he/she would receive 1 point for Item 4). Apart from Item 4, there were also two more items involving this marker, namely Item 6 and Item 10. Then, we calculated the score for the possessive marker as the proportion of the score that the child received out of the total score that he/she could possibly obtain (in this case, the maximum score was 3). The full list of items and details of rescoring are shown in Tables S1 and S2.

### 2.4 Analyses

First, we evaluated acquisition sequences for both the TD and autism groups. The mean score for each grammatical marker was calculated separately by time point and diagnostic group. Next, we validated the acquisition order in the TD group by comparing their scores with those of the TD children reported in Huang et al. (2022) using Pearson correlation tests. Then, we analyzed the acquisition order within the autism group. Specifically, linear regression analyses were conducted with marker type (e.g., negation: *mei*, *bu*, *bie*) as the predictor, and score as the outcome variable, while controlling for the children's age due to considerable variability within each time point. These analyses were conducted separately for each time point.

Second, we used linear mixed‐effects models to compare marker scores across the three time points and between two diagnostic groups, controlling for demographic covariates accordingly. Then, we compared the acquisition orders between the TD and autism groups. ANCOVA analyses were conducted to calculate means adjusted for demographic covariates, and Pearson correlation tests were used to assess the relationship between the adjusted means of the two groups across all 14 markers.

The third goal was to explore the influence of autism severity and initial language ability. Autistic children were divided into subgroups based on their ADOS CSS (i.e., ADOS‐H and ADOS‐L) and the MSEL verbal IQ (i.e., Verbal‐H and Verbal‐L), respectively. Then Pearson correlation tests and MANCOVA analyses were performed to compare their developmental sequences and the scores of 14 markers between subgroups while controlling for demographic covariates.

## 3 Results

### 3.1 Acquisition Sequences

The descriptive statistics for the grammatical marker scores are presented in Table 3. We found a strong correlation between the scores of our TD group and those of the TD children reported in Huang et al. (2022) (*r* = 0.85, *p* < 0.001, *N* = 14). Thus, we replicated the typical developmental sequence of grammatical markers in Mandarin‐speaking children.

**TABLE 3:** Scores and acquisition orders for the autism and TD groups.

Marker	Autism group									TD group		T1 versus T2 versus T3	Autism versus TD
	T1			T2			T3						
	M	SD	Rank	M	SD	Rank	M	SD	Rank	M	SD		
Neg_mei	0.75	0.44	1	0.88	0.33	1	0.94	0.25	1	0.96	0.19	T1 < T2^a^, T1 < T3^b^	T1 < TD^c^
Neg_bu	0.55	0.37	2	0.77	0.34	1.5	0.88	0.25	1.5	0.82	0.30	T1 < T2^c^, T1 < T3^c^	T1 < TD^b^
Possessive	0.44	0.43	2.5	0.71	0.37	2	0.91	0.26	1.5	0.69	0.39	T1 < T2^c^, T1 < T3^c^	T1 < TD^a^, T3 > TD^a^
Asp_le	0.39	0.49	3	0.55	0.5	3	0.84	0.37	1.5	0.67	0.47	T1 < T2^a^, T1 < T3^c^, T2 < T3^b^	T1 < TD^b^
RVC	0.38	0.24	3	0.53	0.23	3	0.69	0.21	2.5	0.53	0.29	T1 < T2^c^, T1 < T3^c^, T2 < T3^b^	T1 < TD^b^
Classifier	0.36	0.45	3	0.62	0.45	2.5	0.88	0.31	1.5	0.56	0.47	T1 < T2^c^, T1 < T3^c^, T2 < T3^a^	T3 > TD^b^
Modal	0.35	0.3	3	0.57	0.3	3	0.75	0.3	2	0.57	0.38	T1 < T2^c^, T1 < T3^c^, T2 < T3^b^	T1 < TD^c^
SFP	0.26	0.24	4	0.42	0.27	4	0.61	0.25	2.5	0.53	0.32	T1 < T2^c^, T1 < T3^c^, T2 < T3^c^	T1 < TD^c^
Adverb	0.18	0.32	4.5	0.36	0.39	4.5	0.61	0.38	2.5	0.52	0.44	T1 < T2^c^, T1 < T3^c^, T2 < T3^b^	T1 < TD^c^
Asp_yao	0.1	0.3	5	0.3	0.46	4.5	0.53	0.51	3	0.46	0.50	T1 < T2^b^, T1 < T3^c^	T1 < TD^c^
Complex_Clause	0.09	0.23	5.5	0.21	0.35	5	0.38	0.42	3.5	0.33	0.41	T1 < T3^c^	T1 < TD^c^
Neg_bie	0.08	0.27	5.5	0.18	0.39	5	0.34	0.48	4	0.26	0.44	T1 < T3^b^	T1 < TD^a^
Asp_guo	0.07	0.25	5.5	0.2	0.4	5	0.56	0.5	2.5	0.48	0.50	T1 < T3^c^, T2 < T3^c^	T1 < TD^c^, T2 < TD^b^
Asp_zheng	0.06	0.23	5.5	0.15	0.36	5.5	0.38	0.49	4	0.29	0.45	T1 < T3^c^, T2 < T3^b^	T1 < TD^c^

*Note:* Markers were ranked based on scores, with a higher rank indicating earlier acquisition. Markers with statistically similar scores share the same rank. A rank difference of 1 or greater indicates a statistically significant difference between the markers.

Abbreviations: Asp = aspect; M = mean; Neg = negation; RVC = resultative verb compound; SD = standard deviation; SFP = sentence final particle.

^a^ *p* < 0.05.

^b^ *p* < 0.01.

^c^ *p* < 0.001.

We also observed a relatively consistent acquisition sequence in the autism group. The results are reported by grammatical category (see also Figure S1).

*Negation mei, bu, bie*: At Time 1, linear regression analyses showed that there was a significant main effect of marker type (*β* = −0.34, SE = 0.03, *t*(264) = −12.04, *p* < 0.001), although the children's age was controlled. Pairwise comparisons (with Bonferroni corrections) showed that among these three Mandarin negative markers, the score for marker *mei* was the highest (*p* < 0.001), followed by marker *bu* (*p* = 0.001) and marker *bie* (*p* < 0.001). At Time 2, there was also a significant main effect of marker type (*β* = 0.35, SE = 0.03, *t*(192) = 10.45, *p* < 0.001). Further pairwise comparisons (with Bonferroni corrections) showed that *mei* and *bu* had significantly higher scores than *bie* (*ps* < 0.001). A similar pattern was also found at Time 3. We found a main effect of marker type (*β* = 0.30, SE = 0.05, *t*(93) = 6.56, *p* < 0.001), with higher scores for *mei* and *bu* than for *bie* (with Bonferroni corrections, *ps* < 0.001). Together, the results suggested that marker *mei* was acquired earlier than marker *bu*, while *bie* was acquired relatively later.

*Aspect yao, le, guo, zheng*: At Time 1, after controlling for the children's age, we found a main effect of marker type (*β* = −0.03, SE = 0.01, *t*(353) = −2.39, *p* = 0.018). We further conducted pairwise comparisons with Bonferroni corrections and found that the score for aspect marker *le* was significantly higher than for the other three aspect markers (*yao*, *guo*, and *zheng*, *ps* < 0.001). Similar main effects of marker type were also found at Time 2 (*β* = 0.07, SE = 0.02, *t*(257) = 3.75, *p* < 0.001) and Time 3 (*β* = 0.06, SE = 0.03, *t*(125) = 2.27, *p* = 0.025). The score for aspect marker *le* was significantly higher than for the other three aspect markers at Time 2 (*ps* < 0.01). At Time 3, the score for *le* was higher than for *yao* and *zheng* (*ps* < 0.05), but comparable with that for *guo* (*p* = 0.053). The data showed that aspect marker *le* was acquired earlier than the other three aspect markers (*yao*, *guo*, and *zheng*).

*Modals, RVC, adverbs*: After controlling for the children's age, the main effects of marker type were found at Time 1 (*β* = −0.08, SE = 0.02, *t*(264) = −3.57, *p* < 0.001) and Time 2 (*β* = 0.11, SE = 0.03, *t*(192) = 3.85, *p* < 0.001), but not at Time 3 (*p* = 0.055). At both Time 1 and Time 2, the scores of RVC and modals were significantly higher than those of adverbs (*ps* < 0.01, with Bonferroni corrections), suggesting that RVC and modals were acquired earlier than adverbs.

*SFPs and complex clauses*: The main effects of marker type were found at Time 1 (*β* = −0.09, SE = 0.02, *t*(175) = −4.75, *p* < 0.001), Time 2 (*β* = 0.1, SE = 0.03, *t*(127) = 3.6, *p* < 0.001), and Time 3 (*β* = 0.12, SE = 0.04, *t*(61) = 2.69, *p* = 0.009). The score for SFPs was significantly higher than for complex clauses across three time points, suggesting that SFPs were acquired relatively earlier.

*Possessives and classifiers*: No effect of marker type was found at three time points (*ps* > 0.05), showing similar developmental patterns for these two categories of markers.

### 3.2 Comparisons Across Time Points and Between Diagnostic Groups

*Time 1* versus *Time 2* versus *Time 3*: Separate linear‐mixed effects models were used to examine the effect of time point on autistic children's performance of each Mandarin grammatical marker, while controlling for the covariables including sex, maternal education, and family income. As shown in Table 3, the scores for all markers significantly increased across the three time points (*ps* < 0.05), suggesting that these autistic children's mastery of grammatical markers had developed over time.

*TD* versus *Autism*: We next compared scores between the TD and autism groups using linear‐mixed effects models, controlling for participant sex, maternal education, and family income (Table 3). The results indicated that at Time 1, autistic children's scores were significantly lower than that of TD children for 13 out of the 14 grammatical markers (*ps* < 0.05). The only exception was classifiers, for which the two groups showed comparable performance (*p* > 0.05). At Time 2, there were no significant differences between groups for 13 markers (*ps* > 0.05), with one exception of the aspect marker *guo*, for which the autism group scored significantly lower (*p* < 0.01). At Time 3, the autism group had significantly higher scores than the TD group for two markers: possessive *de* (*p* < 0.05) and classifiers (*p* < 0.01). Overall, these results suggested that the grammatical marker developmental level of the autism group was generally lower than that of the TD group at Time 1. However, by Time 2 and Time 3, it had largely caught up to the level of the TD group, with two notable exceptions: the aspect marker *guo* developed more slowly, while classifiers developed more rapidly in the autism group.

**FIGURE 1:**
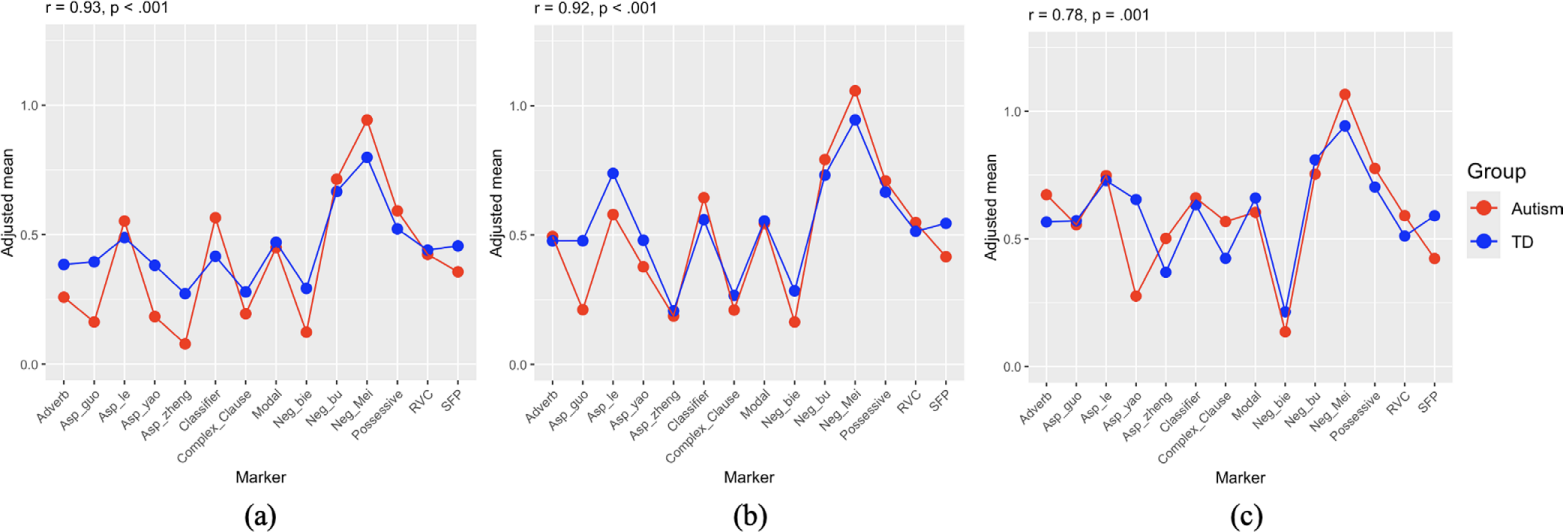
Comparison of adjusted means between the TD group and the autism group at Time 1 (a), Time 2 (b), and Time 3 (c).

Then, we compared the acquisition orders of grammatical markers between the TD and autism groups (Figure 1). For each marker, we performed ANCOVA analyses to calculate adjusted means for both groups, controlling for the participants' age, language ability (indexed by vocabulary production and sentence complexity raw scores), sex, maternal education, and family income. We then conducted Pearson correlation tests to examine the relationship between the adjusted means of the autism and TD groups across all 14 markers. The results showed strong and significant correlations between the two groups at both Time 1 (*r* = 0.93, *p* < 0.001, *N* = 14) and Time 2 (*r* = 0.92, *p* < 0.001, *N* = 14). Although the correlation at Time 3 decreased slightly, it remained very strong (*r* = 0.78, *p* < 0.001, *N* = 14). These findings suggested that autistic children's acquisition orders were similar to those of TD children even after controlling for participant age, language ability, sex, maternal education, and family income.

### 3.3 The Effect of Autism Severity and Initial Language Ability

We further explored if the two potential factors, namely autism severity and initial language ability, would influence autistic children's grammatical marker developmental patterns.

To explore the effect of autism severity, the score for each grammatical marker was calculated separately for the ADOS‐H and ADOS‐L subgroups (Table 4). The relationships between these two subgroups were evaluated across three time points, controlling for age, sex, maternal education, family income, children's current (indexed by vocabulary production and sentence complexity raw scores) and initial (indexed by Mullen verbal IQ) language abilities. The results of the Pearson correlation tests revealed strong positive correlations between the two subgroups at Time 1 (*r* = 0.98, *p* < 0.001, *N* = 14), Time 2 (*r* = 0.96, *p* < 0.001, *N* = 14), and Time 3 (*r* = 0.93, *p* < 0.001, *N* = 14). MANCOVA analyses revealed significant subgroup differences in the scores for 14 markers at Time 1 (F (14, 58) = 1.92, *p* = 0.043), but not at Time 2 and Time 3 (*p* > 0.05).

**TABLE 4:** Scores for grammatical markers grouped by autism severity.

	Time 1		Time 2		Time 3		TD
	ADOS_H	ADOS_L	ADOS_H	ADOS_L	ADOS_H	ADOS_L	
CSS (mean)	8.48	5.94	8.29	5.88	8.17	5.8	
*N*	23	62	14	51	6	25	
Neg_mei	0.78	0.88	0.93	0.88	0.9	0.92	0.96
Neg_bu	0.63	0.74	0.81	0.76	0.78	0.86	0.82
Possessive	0.51	0.57	0.64	0.73	0.76	0.88	0.69
RVC	0.35	0.51	0.55	0.53	0.54	0.69	0.53
Asp_le	0.39	0.47	0.50	0.57	0.58	0.84	0.67
Classifier	0.39	0.56	0.61	0.63	0.65	0.84	0.56
Modal	0.39	0.57	0.63	0.56	0.58	0.74	0.57
SFP	0.28	0.44	0.45	0.41	0.42	0.62	0.53
Adverb	0.22	0.39	0.43	0.35	0.36	0.61	0.52
Asp_yao	0.13	0.24	0.29	0.31	0.33	0.56	0.46
Complex_Clause	0.07	0.26	0.29	0.20	0.2	0.38	0.33
Neg_bie	0.09	0.29	0.29	0.14	0.13	0.28	0.26
Asp_guo	0.09	0.24	0.21	0.20	0.19	0.56	0.48
Asp_zheng	0.09	0.18	0.21	0.14	0.15	0.36	0.29

Abbreviations: Asp = Aspect; CSS = Calibrated Severity Score; Neg = Negation; RVC = resultative verb compound; SFP = sentence final particle.

Regarding the factor of initial language ability, the score for each grammatical marker was calculated for the Verbal‐H and Verbal‐L subgroups (Table 5). After controlling for the participants' age, sex, maternal education, family income, current language ability (indexed by vocabulary production and sentence complexity raw scores) and autism severity (indexed by ADOS‐2 CSS), the results of the Pearson correlation tests suggested significant correlations between the Verbal‐H and Verbal‐L subgroups at Time 1 (*r* = 0.94, *p* < 0.001, *N* = 14), Time 2 (*r* = 0.95, *p* < 0.001, *N* = 14), and Time 3 (*r* = 0.81, *p* < 0.001, *N* = 14). MANCOVA analyses revealed significant subgroup differences in the scores for 14 markers at Time 1 (F (14, 58) = 22.16, *p* < 0.001), Time 2 (F (14, 39) = 10.46, *p* < 0.001), and Time 3 (F (14, 5) = 11.09, *p* = 0.007).

**TABLE 5:** Scores for grammatical markers grouped by initial language ability.

	Time 1		Time 2		Time 3		TD
	Verbal‐H	Verbal‐L	Verbal‐H	Verbal‐L	Verbal‐H	Verbal‐L	
Verbal IQ (mean)	86.12	44.57	85.83	45.54	88.39	47.92	
*N*	34	51	30	35	18	13	
Neg_mei	0.82	0.71	0.93	0.86	1.00	0.85	0.96
Neg_bu	0.64	0.51	0.82	0.73	0.90	0.85	0.82
Possessive	0.57	0.35	0.73	0.69	0.93	0.87	0.69
Asp_le	0.47	0.35	0.60	0.51	0.89	0.85	0.53
RVC	0.48	0.31	0.57	0.50	0.70	0.67	0.67
Classifier	0.54	0.25	0.73	0.53	0.94	0.77	0.56
Modal	0.42	0.31	0.62	0.53	0.82	0.65	0.57
SFP	0.33	0.22	0.48	0.38	0.67	0.54	0.53
Adverb	0.28	0.12	0.49	0.27	0.72	0.46	0.52
Asp_yao	0.21	0.04	0.37	0.26	0.56	0.46	0.46
Complex_Clause	0.16	0.05	0.22	0.21	0.53	0.15	0.33
Neg_bie	0.09	0.08	0.20	0.14	0.44	0.15	0.26
Asp_guo	0.15	0.02	0.27	0.14	0.67	0.38	0.48
Asp_zheng	0.09	0.04	0.23	0.09	0.56	0.15	0.29

Abbreviations: Asp = aspect; Neg = negation; RVC = resultative verb compound; SFP = sentence final particle.

## 4 Discussion

This study aimed to systematically investigate the developmental patterns of a wide range of Mandarin grammatical markers in Chinese autistic children. We used a parent reporting approach and a grammatical marking rescoring method adopted from Huang et al. (2022) to analyze children's mastery of each grammatical marker. Our findings showed that autistic children acquired Mandarin grammatical markers in a typical sequence, though their development was delayed. Furthermore, factors including autism severity and initial language ability did not alter this developmental pattern. These results supported the quantitative difference hypothesis.

### 4.1 Developmental Pattern of Mandarin Grammatical Markers

Our first research question was about the pattern of autistic children acquiring Mandarin grammatical markers. The findings showed a clear acquisition order which was stable across time points. Specifically, we found that negative *mei*, *bu*, and possessive *de* were acquired the earliest, followed by aspect *le*, resultative verb compound, classifiers, modals, sentence final particles, and adverbs. Aspect *yao*, complex clauses, negative *bie*, aspect *guo*, and aspect *zheng* were the latest‐acquired markers. Moreover, though all the markers developed across time points, those markers that ranked higher (i.e., had a higher degree of mastery) at Time 1 always ranked higher at Time 2 and Time 3. The evidence revealed that autistic children acquired Mandarin markers sequentially.

More importantly, after controlling for the participants' age, sex, maternal education, family income, and language ability, the acquisition orders of grammatical markers observed in autistic children were highly associated with those of TD children, indicating similar acquisition sequences between the two diagnostic groups. It was worth noting that although the correlation decreased slightly at Time 3, it remained very strong. This subtle decline might reflect a subsequent stage of grammatical marker development, given that the language ability of the autism group at Time 3 was slightly (but not significantly) higher than that of TD children.

Our study also found a delay in autistic children's development, while they had eventually caught up to the level of the TD group for all grammatical markers. Specifically, at Time 1, autistic children's scores were lower than those of TD children for 13 out of the 14 grammatical markers, with classifiers being the only exceptions. However, the scores became comparable at Time 2, with one exception of the experiential aspect marker *guo*, for which the autism group had significantly lower scores. At Time 3, the autism group's scores for all markers including the marker *guo* were comparable to or even higher than those of TD children. These patterns indicated that classifiers developed relatively faster in the autism group, while the experiential aspect marker *guo* developed more slowly. These findings aligned with those of previous studies reporting that autistic children produce the aspect marker *guo* less frequently than TD children, but produce classifiers at similar levels (Su et al. 2018). Autistic children's particular delay in the acquisition of the marker *guo* could be attributed to broader pragmatic deficits in autism, including a reduced reference to past or nonpresent events (Su and Naigles 2023). It is important to note that while the autism group's scores for all markers became comparable to those of the TD control group by Time 3, this does not mean that they were at an age‐appropriate level because the autistic children were significantly older than the TD children. Our results suggest a significant delay in the development of grammatical markers among autistic children.

In general, the current study indicates that autistic children acquire Mandarin grammatical markers in a typical sequence but at a slower rate, which supports the quantitative difference hypothesis.

### 4.2 The Role of Autism Severity and Initial Language Ability

This study found that autism severity does not change autistic children's pattern of grammatical marker development. The acquisition orders between ADOS‐H and ADOS‐L groups were strongly correlated across all three time points, after controlling for the participants' age, sex, maternal education, family income, and both initial and current language abilities. This result is consistent with previous findings that the severity of autism symptoms does not predict autistic children's language development (e.g., So and Song 2023; Nevill et al. 2019). Nevill et al. (2019) further claimed that this might reflect the nature of the ADOS‐2, which is intended to assess autism symptoms independently from participants' language level. However, it is worth noting that our participants were attending a supporting program aimed at enhancing autistic children's social communication outcomes, and the effectiveness of this program has been reported (Wang et al. 2026; Wong et al. 2025). Furthermore, the age of 4 to 6 years old was found to be a crucial period for autistic preschoolers' Mandarin grammatical marker development (Li et al. 2023), and this was also the period when our subjects participated in the program. Thus, it is possible that our program helped those autistic children maintain the typical sequential developmental pattern. In other words, without support, those children with a higher level of autism spectrum‐related symptoms might alter their acquisition order, which should be further investigated.

In addition, our results also show that the developing pattern is not affected by the initial language ability. Though the Verbal‐L group was significantly more delayed than the Verbal‐H group, the acquisition orders between the two subgroups were highly correlated after controlling for the participants' age, sex, maternal education, family income, autism severity, and current language ability. This supported the quantitative difference hypothesis. It is also important to note that although the autistic participants were divided into two language groups, all autistic children in this study had a relatively limited initial language ability: the average score for the Mullen verbal IQ was 68.2 (SD = 29.4), and more than half (60%, 51 out of 85) of the children were assigned to the Verbal‐L group. Given the well‐documented heterogeneity in expressive language ability among autistic children (e.g., Tek et al. 2014; Wittke et al. 2017), the current sample might not be fully representative of the broader autism population. Nevertheless, the results of the current study reveal that even among participants with such low initial language abilities, there were no qualitative differences identified in regard to grammatical marker development.

Taken together, the results suggested that the acquisition orders of autistic children regardless of autism severity and initial language ability are highly correlated with that of TD children, which further supports the quantitative difference hypothesis. This conclusion has also been supported by previous research in various linguistic domains, such as lexicon (Ellis Weismer et al. 2011), morphosyntax (Su and Naigles 2023), and sentence processing (Zhou, Zhan, and Ma 2019). These findings shed light on the distinction between linguistic competence and performance in the Universal Grammar framework (Chomsky 1965). They imply that autistic children's (including those having limited expressive language) underlying linguistic knowledge or competence is intact, while their performance is different from that of TD children (e.g., developmental delay). Thus, perhaps it is more appropriate to view the difference between TD and autistic children as a developmental continuity (Zhou, Zhan, and Ma 2019).

### 4.3 Clinical Implications and Limitations

The results of this study have important implications for clinical practice. We observed a significant delay in the Mandarin grammatical marker development among autistic children. Nevertheless, their grammatical marker skills improved along with general language development, even in the absence of intervention targeting morphosyntax. This implies that early intervention aimed at facilitating general language ability may be of primary importance. Notably, the autistic children in this study with a lower initial language ability showed greater delays across three time points, indicating that they are likely to have more challenges in grammatical marker development. Therefore, they may benefit from direct morphosyntactic intervention. Moreover, our findings suggest that autistic children acquire Mandarin grammatical markers in a typical developmental sequence, which informs the design of more targeted and efficient intervention strategies. For example, following the traditional structured hierarchical approach (Fogle 2019), clinicians could design morphosyntactic intervention beginning with the earliest‐acquired markers (i.e., negative *mei*, *bu*, and possessive *de*). These markers may be easier for autistic children; thereby, children would be more motivated and show greater overall improvement.

Several limitations of this study should be considered when interpreting the findings. First, this study measured children's mastery of grammatical markers using the CCDI‐P, which is a parent report instrument. Therefore, we cannot exclude the influence of parental subjectivity on the accuracy of reporting their child's language ability. Furthermore, instead of being asked for specific grammatical markers, parents were asked to select the utterance that most matched their child's language level. In other words, we did not directly measure children's knowledge of each grammatical marker. Additionally, most autistic participants were older than the standardized age range (16–30 months) of the CCDI‐P Words and Sentences form, which might limit the tool's sensitivity in capturing these children's full abilities. Future research is encouraged to combine parent reports with other assessments (e.g., children's spontaneous speech, elicitation tasks) to compensate for the shortages. Moreover, since the CCDI‐P consists of a closed set of items, the grammatical markers included are limited. Further investigation is needed with a more comprehensive marker list. In addition, our participants were taken from a larger longitudinal support program. Due to the loss of follow‐ups, there is an imbalance in the data size at each time point. Well‐designed longitudinal studies addressing this limitation are highly recommended in future. Also, the potential influence of the supporting program itself on the development of grammatical markers was not accounted for, as the intervention was not specifically targeted at morphosyntax, and our autistic children showed qualitative similarities to TD children at the baseline. Nevertheless, we cannot rule out the possibility that without such support, autistic children might not maintain the typical acquisition order. Finally, though the participants in this study had a relatively more limited language ability, for whom we only found quantitative rather than qualitative differences, they still could not represent the entire autistic population due to the huge heterogeneity. Future studies that include autistic children with a greater variety of language abilities are encouraged to verify the present findings.

## 5 Conclusion

Using a parent reporting approach and a longitudinal study design, this study investigated grammatical marker development in Mandarin‐speaking autistic children across three time points. We found that although there was a delay, autistic children acquired the markers in a typical order. Additionally, the two factors, namely children's autism severity and initial language ability, did not alter this developmental pattern. Follow‐up results suggested that the autistic children had largely caught up to the level of the TD children for all the target markers. Taken together, these findings support the quantitative difference hypothesis and imply intact linguistic competence in autistic children.

## Funding

This work was supported by the Research Grants Council (HKSAR) (C4024‐21G) and the Shenzhen Natural Science Foundation Grant (JCYJ20220531103803009).

## Ethics Statement

The study was approved by the Chinese University of Hong Kong Research Ethics Committee (CREC Ref. No. 2021.523‐T).

## Consent

The authors have nothing to report.

## Conflicts of Interest

The authors declare no conflicts of interest.

## Supporting information


**Table S1:** Items and the coding of grammatical markers in CCDI‐P (adapted from Huang et al. 2022).
**Table S2:** Items for grammatical marking rescoring (adapted from Huang et al. 2022).
**Figure S1:** Score comparison across grammatical categories and three time points.

## Data Availability

The data that support the findings of this study are available from the corresponding author upon reasonable request.
