# Supplementary material for: Quantitative but Not Qualitative Differences: A Longitudinal Analysis of Grammatical Marker Development in Mandarin‐Speaking Autistic Children
Source: Autism Res. 2026 Feb 7;19(4):e70195. doi: 10.1002/aur.70195 (PMC13087843; doi:10.1002/aur.70195)
Supplement: Supplementary file 1 — Table S1: Items and the coding of grammatical markers in CCDI‐P (adapted from Huang et al. 2022). Table S2: Items for grammatical marking rescoring (adapted from Huang et al. 2022). Figure S1: Score comparison across grammatical categories and three time points. [file AUR-19-0-s001.docx]

**Supplementary Information**

Table S1. Items and the coding of grammatical markers in CCDI-P (adapted from Huang et al., 2022)

| **Item** | **Target Expression** | **Grammatical Coding** | **Expression Options** | **Rescoring Variable Name** | |
| --- | --- | --- | --- | --- | --- |
| 1 | Express something is gone (表示东西不见了) | Cannot say | 不会说 | | 01_0 |
|  |  | NEG-mei (没) | 没 (gone) | | 01_1 |
|  |  | NEG-mei (没) + le | 没了 (gone) | | 01_2 |
|  |  | S+NEG-mei (没) + SFP-le | 车没(有)了 (car is gone) | | 01_3 |
|  |  |  |  | |  |
| 2 | Express desire  (表示愿望) | Cannot say | 不会说 | | 02_0 |
|  |  | O | 球 (ball) | | 02_1 |
|  |  | V-yao (要) | 要 (want) | | 02_2 |
|  |  | V-yao (要) + O | 要球 (want ball) | | 02_3 |
|  |  |  |  | |  |
| 3 | Express desire  (表示愿望) | Cannot say | 不会说 | | 03_0 |
|  |  | V + O | 去外外 (go outside) | | 03_1 |
|  |  | V + RVC | 出去 (go out) | | 03_2 |
|  |  | Modal-yao (要) + V + RVC | 要出去 (want to go out) | | 03_3 |
|  |  | S + Modal-yao (要) + V + RVC | 我要出去 (I want to go out) | | 03_4 |
|  |  |  |  | |  |
| 4 | Express possession (表示所属) | Cannot say | 不会说 | | 04_0 |
|  |  | N + N | 宝宝车 (baby car) | | 04_1 |
|  |  | N + Poss-de (的) + N | 宝宝的车 (baby’s car) | | 04_2 |
|  |  | Pronoun + poss-de (的) + N | 我的车 (my car) | | 04_3 |
|  |  |  |  | |  |
| 5 | Express desire  (表示愿望) | Cannot say | 不会说 | | 04_0 |
|  |  | V + O | 喝水 (drink water) | | 05_1 |
|  |  | S + V + O | 我喝水 (I drink water) | | 05_2 |
|  |  | Modal-yao (要) + V + O | 要喝水 (want to drink water) | | 05_3 |
|  |  | S+Modal-yao (要) + V + O | 我要喝水 (I want to drink water) | | 05_4 |
|  |  |  |  | |  |
| 6 | Express possession (表示所属) | Cannot say | 不会说 | | 06_0 |
|  |  | N + N | 妈妈车 (mom car) | | 06_1 |
|  |  | N + Poss-de (的) + N | 妈妈的车 (mom’s car) | | 06_2 |
|  |  |  |  | |  |
| 7 | Express future events (讲将要发生的事) | Cannot say | 不会说 | | 07_0 |
|  |  | S + V | 球掉 (ball fall) | | 07_1 |
|  |  | S+ASP-future-yao (要) +V | 球要掉 (ball will fall) | | 07_2 |
|  |  |  |  | |  |
| 8 | If cannot do something (如果不能做某事) | Cannot say | 不会说 | | 08_0 |
|  |  | NEG-bu (不) | 不 (no) | | 08_1 |
|  |  | NEG-bu (不) + V | 不拿 (no pick) | | 08_2 |
|  |  | NEG-bu (不) + V + RVC | 不拿起来 (no pick up) | | 08_3 |
|  |  | V+NEG-bu (不) + RVC | 拿不起来 (can’t pick up) | | 08_4 |
|  |  |  |  | |  |
| 9 | Asking someone to do something (如果要人为他做某事) | Cannot say | 不会说 | | 09_0 |
|  |  | V | 讲 (tell) | | 09_1 |
|  |  | S + V | 妈妈讲 (mom tell) | | 09_2 |
|  |  | S + V + O | 妈妈讲故事 (mom tell story) | | 09_3 |
|  |  | V + O + O | 讲故事宝宝 (tell story baby) | | 09_4 |
|  |  | S1 + Prep-gei (给) + O1 + V + O2 | 妈妈给宝宝讲故事 (mom tell baby story) | | 09_5 |
|  |  |  |  | |  |
| 10 | Express self ownership (表示东西是自己的) | Cannot say | 不会说 | | 10_0 |
|  |  | Pronoun-wo (我) + Poss-de (的) | 我的 (mine) | | 10_1 |
|  |  | S + Pronoun-wo (我) + Poss-de (的) | 那我的 (that mine) | | 10_2 |
|  |  | S + V + Pronoun-wo (我) + Poss-de (的) | 那是我的 (that is mine) | | 10_3 |
|  |  |  |  | |  |
| 11 | Express wanting something (讲想要的东西) | Cannot say | 不会说 | | 11_0 |
|  |  | S + V | 宝宝要 (baby want) | | 11_1 |
|  |  | S + V + O | 宝宝要球 (baby want ball) | | 11_2 |
|  |  | S + Modal-xiang (想) + V + O | 宝宝想要球 (baby want ball) | | 11_3 |
|  |  |  |  | |  |
| 12 | If need to do more (如果要再做某件事) | Cannot say | 不会说 | | 12_0 |
|  |  | V + Adv-dian (点) | 吃点 (eat some) | | 12_1 |
|  |  | Adv-zai (再) + V + Adv-dian (点) | 再吃点 (eat some more) | | 12_2 |
|  |  |  |  | |  |
| 13 | Express past events (讲过去发生的事) | Cannot say | 不会说 | | 13_0 |
|  |  | S + V | 哥哥摔 (brother fall) | | 13_1 |
|  |  | S + V + RVC | 哥哥摔跤 (brother fall) | | 13_2 |
|  |  | S+V+Asp-le (了) | 哥哥摔了 (brother fell) | | 13_3 |
|  |  | S + V + RVC + Asp-le (了) | 哥哥摔跤了 (brother fell) | | 13_4 |
|  |  |  |  | |  |
| 14 | Express ongoing events (讲正在发生的事) | Cannot say | 不会说 | | 14_0 |
|  |  | S + V | 娃娃哭 (baby cry) | | 14_1 |
|  |  | S + V + SFP-le (了) | 娃娃哭了 (baby is crying) | | 14_2 |
|  |  |  |  | |  |
| 15 | If don’t want something to happen (如果不想有某事发生) | Cannot say | 不会说 | | 15_0 |
|  |  | NEG-bu (不)+V | 不动 (no move) | | 15_1 |
|  |  | NEG-bu (不)+Modal-xu (许)/neng (能) +V | 不许/不能动 (not allow to/cannot move) | | 15_2 |
|  |  | NEG-bie (别)+V | 别动 (don’t move) | | 15_3 |
|  |  |  |  | |  |
| 16 | Express past events (讲发生过的事) | Cannot say | 不会说 | | 16_0 |
|  |  | S+V | 哥哥打 (brother hit) | | 16_1 |
|  |  | S+V+O | 哥哥打我 (brother hit me) | | 16_2 |
|  |  | S+V+O+SFP-le | 哥哥打我了 (brother has hit me) | | 16_3 |
|  |  |  |  | |  |
| 17 | If want something to happen again (如果又发生某事) | Cannot say | 不会说 | | 17_0 |
|  |  | S + V | 他要 (he want) | | 17_1 |
|  |  | S + Adv-hai (还) + V | 他还要 (he want again) | | 17_2 |
|  |  |  |  | |  |
| 18 | If do not want others to do something (如果不想让他人做某事) | Cannot say | 不会说 | | 18_0 |
|  |  | NEG-bu (不) + Modal-yao (要) + V + O | 不要看书 (don’t read books) | | 18_1 |
|  |  | S+NEG-bu (不) + Modal-yao (要) + V + O | 你不要看书 (you don’t read books) | | 18_2 |
|  |  | S1+NEG-bu (不)+Modal-yao (要)+S2+V+O | 我不要你看书 (I don’t want you to read books) | | 18_3 |
|  |  |  |  | |  |
| 19 | If do not want something to happen (如果不希望某事发生) | Cannot say | 不会说 | | 19_0 |
|  |  | NEG-bu (不) + V + O | 不洗小娃娃 (no wash toy) | | 19_1 |
|  |  | NEG-bu (不) + Modal-neng (能) + V + O | 不能洗小娃娃 (cannot wash toy) | | 19_2 |
|  |  |  |  | |  |
| 20 | If something happened again (如果又发生某事) | Cannot say | 不会说 | | 20_0 |
|  |  | S + V + SFP-le | 他来了 (he has come) | | 20_1 |
|  |  | S + Adv-you (又) + V | 他又来 (he come again) | | 20_2 |
|  |  | S + Adv-you (又) + V + SFP-le | 他又来了 (he has come again) | | 20_3 |
|  |  |  |  | |  |
| 21 | Express ongoing events (讲正在发生的事) | Cannot say | 不会说 | | 21_0 |
|  |  | S+V+O | 我吃饭 (I eat) | | 21_1 |
|  |  | S + V + O + SFP-ne (呢) | 我吃饭呢！(I’m eating) | | 21_2 |
|  |  | S+Asp-zheng (正) + V + O + SFP-ne (呢) | 我正吃饭呢！(I’m eatinge) | | 21_3 |
|  |  |  |  | |  |
| 22 | Ask questions (提问) | Cannot say | 不会说 | | 22_0 |
|  |  | Prounoun-zhe (这) | 这？(this?) | | 21_1 |
|  |  | Prounoun-zhe (这) + SFP-ne (呢) | 这（个）呢？(this?) | | 21_2 |
|  |  | Q-shenme (什么) | 什么？(what?) | | 21_3 |
|  |  | S + V + Q-shenme (什么) | 这是什么？(what is this?) | | 21_4 |
|  |  |  |  | |  |
| 23 | Express classifiers (讲数量词) | Cannot say | 不会说 | | 23_0 |
|  |  | Num + N | 一人 (one man) | | 23_1 |
|  |  | Num + CL-ge (个) +N | 一个人 (one + CL-ge4 + man = a man) | | 23_2 |
|  |  |  |  | |  |
| 24 | Express classifiers (讲数量词) | Cannot say | 不会说 | | 24_0 |
|  |  | Num + CL-ge (个) + N | 一个车 (one + CL-ge4 + car = one car) | | 24_1 |
|  |  | Num + CL-liang (辆) + N | 一辆车(one + CL-liang4 + car = one car) | | 24_2 |
|  |  |  |  | |  |
| 25 | Express past events (讲以前发生过的事) | Cannot say | 不会说 | | 25_0 |
|  |  | S+V | 我做 (I do) | | 25_1 |
|  |  | S+V+Asp-guo (过) | 我做过 (I did) | | 25_2 |
|  |  |  |  | |  |
| 26 | If not able to do something (如果做不了某事) | Cannot say | 不会说 | | 26_0 |
|  |  | NEG-bu (不) | 不 (no) | | 26_1 |
|  |  | NEG-bu (不) + Modal-hui (会) | 不会 (no + can = cannot) | | 26_2 |
|  |  | S + NEG-bu (不) + Modal-hui (会) | 我不会 (I cannot) | | 26_3 |
|  |  | S + NEG-bu (不) + Modal-hui (会) + V | 我不会做 (I cannot do) | | 26_4 |
|  |  | S + V + NEG-bu (不) + RVC-liao (了) | 我做不了(I cannot do) | | 26_5 |
|  |  |  |  | |  |
| 27 | Asking questions (提问) | Cannot say | 不会说 | | 27_0 |
|  |  | V | 玩？(play?) | | 27_1 |
|  |  | Modal-neng (能) + V | 能玩？(can play?) | | 27_2 |
|  |  | Modal-neng (能) + V + SFP-ma (吗) | 能玩吗？(can play + SFP-ma1) | | 27_3 |

Table S2. Items for grammatical marking rescoring (adapted from Huang et al., 2022)

| **Negation (max = 7)** |  |
| --- | --- |
| **Variable Name** | **Score** |
| 01_1 or 01_2 or 01_3 (mei) | 1 |
| 01_0 | 0 |
| 08_1 or 08_2 or 08_3 or 08_4 (bu) | 1 |
| 08_0 | 0 |
| 15_1 or 15_2 or 15_3 (bu) | 1 |
| 15_0 | 0 |
| 18_1 or 18_2 or 18_3 (bu) | 1 |
| 18_0 | 0 |
| 19_1 or 19_2 (bu) | 1 |
| 19_0 | 0 |
| 26_1 or 26_2 or 26_3 or 26_4 or 26_5 (bu) | 1 |
| 26_0 | 0 |
| 15_3 (bie) | 1 |
| 15_0 or 15_1 or 15_2 | 0 |
|  |  |
| **Modal (max = 7)** |  |
| **Variable Name** | **Score** |
| 03_3 or 03_4 (yao) | 1 |
| 03_0 or 03_1 or 03_2 | 0 |
| 05_3 or 05_4 (yao) | 1 |
| 05_0 or 05_1 or 05_2 | 0 |
| 11_3 (xiang) | 1 |
| 11_0 or 11_1 or 11_2 | 0 |
| 18_1 or 18_2 or 18_3 (yao) | 1 |
| 18_0 | 0 |
| 19_2 (neng) | 1 |
| 19_0 or 19_1 | 0 |
| 27_2 or 27_3 (neng) | 1 |
| 27_0 or 27_1 | 0 |
| 26_2 or 26_3 or 26_4 or 26_5 (hui) | 1 |
| 26_0 or 26_1 | 0 |
|  |  |
| **RVC (max = 3)** |  |
| **Variable Name** | **Score** |
| 03_2 or 03_3 or 03_4 (chuqu) | 1 |
| 03_0 or 03_1 | 0 |
| 08_3 or 08_4 (naqilai) | 1 |
| 08_0 or 08_1 or 08_2 | 0 |
| 26_5 (zuobuliao) | 1 |
| 26_0 or 26_1 or 26_2 or 26_3 or 26_4 | 0 |
|  |  |
| **Aspect (max = 4)** |  |
| **Variable Name** | **Score** |
| 07_2 (yao) | 1 |
| 07_0 or 07_1 | 0 |
| 13_3 or 13_4 (le) | 1 |
| 13_0 or 13_1 or 13_2 | 0 |
| 25_2 (guo) | 1 |
| 25_0 or 25_1 | 0 |
| 21_3 (zheng) | 1 |
| 21_0 or 21_1 or 21_2 | 0 |
|  |  |
| **Possessive (max = 3)** |  |
| **Variable Name** | **Score** |
| 04_2 or 04_3 | 1 |
| 04_0 or 04_1 | 0 |
| 06_2 | 1 |
| 06_0 or 06_1 | 0 |
| 10_1 or 10_2 or 10_3 | 1 |
| 10_0 | 0 |
|  |  |
| **Classifier (max = 2)** |  |
| **Variable Name** | **Score** |
| 23_2 (ge) | 1 |
| 23_0 or 23_1 | 0 |
| 24_1 or 24_2 (liang) | 1 |
| 24_0 | 0 |
|  |  |
| **Adverb (max = 3)** |  |
| **Variable Name** | **Score** |
| 12_2 (zai) | 1 |
| 12_0 or 12_1 | 0 |
| 17_2 (hai) | 1 |
| 17_0 or 17_1 | 0 |
| 20_2 or 20_3 (you) | 1 |
| 20_0 or 20_1 | 0 |
|  |  |
| **Sentence Final Particle (max = 7)** |  |
| **Variable Name** | **Score** |
| 14_2 (le) | 1 |
| 14_0 or 14_1 | 0 |
| 16_3 (le) | 1 |
| 16_0 or 16_1 or 16_2 | 0 |
| 20_1 or 20_3 (le) | 1 |
| 20_0 or 20_2 | 0 |
| 01_2 or 01_3 (le) | 1 |
| 01_0 or 01_1 | 0 |
| 21_2 or 21_3 (ne) | 1 |
| 21_0 or 21_1 | 0 |
| 22_2 (ne) | 1 |
| 22_0 or 22_1 or 22_3 or 22_4 | 0 |
| 27_3 (ma) | 1 |
| 27_0 or 27_1 or 27_2 | 0 |
|  |  |
| **Complex Clauses (max = 2)** |  |
| **Variable Name** | **Score** |
| 09_5 | 1 |
| 09_0 or 09_1 or 09_2 or 09_3 or 09_4 | 0 |
| 18_3 | 1 |
| 18_0 or 18_1 or 18_2 | 0 |


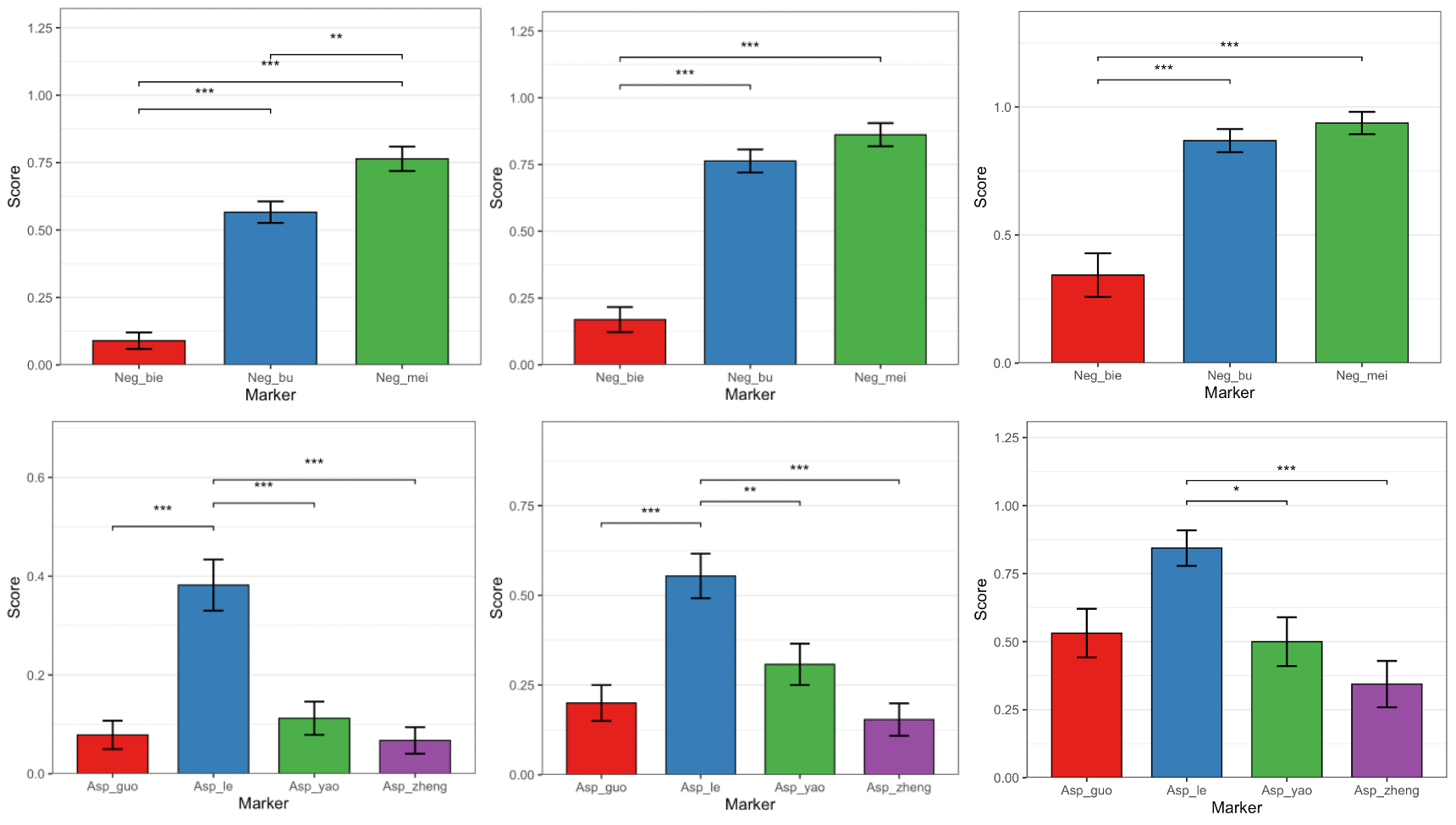


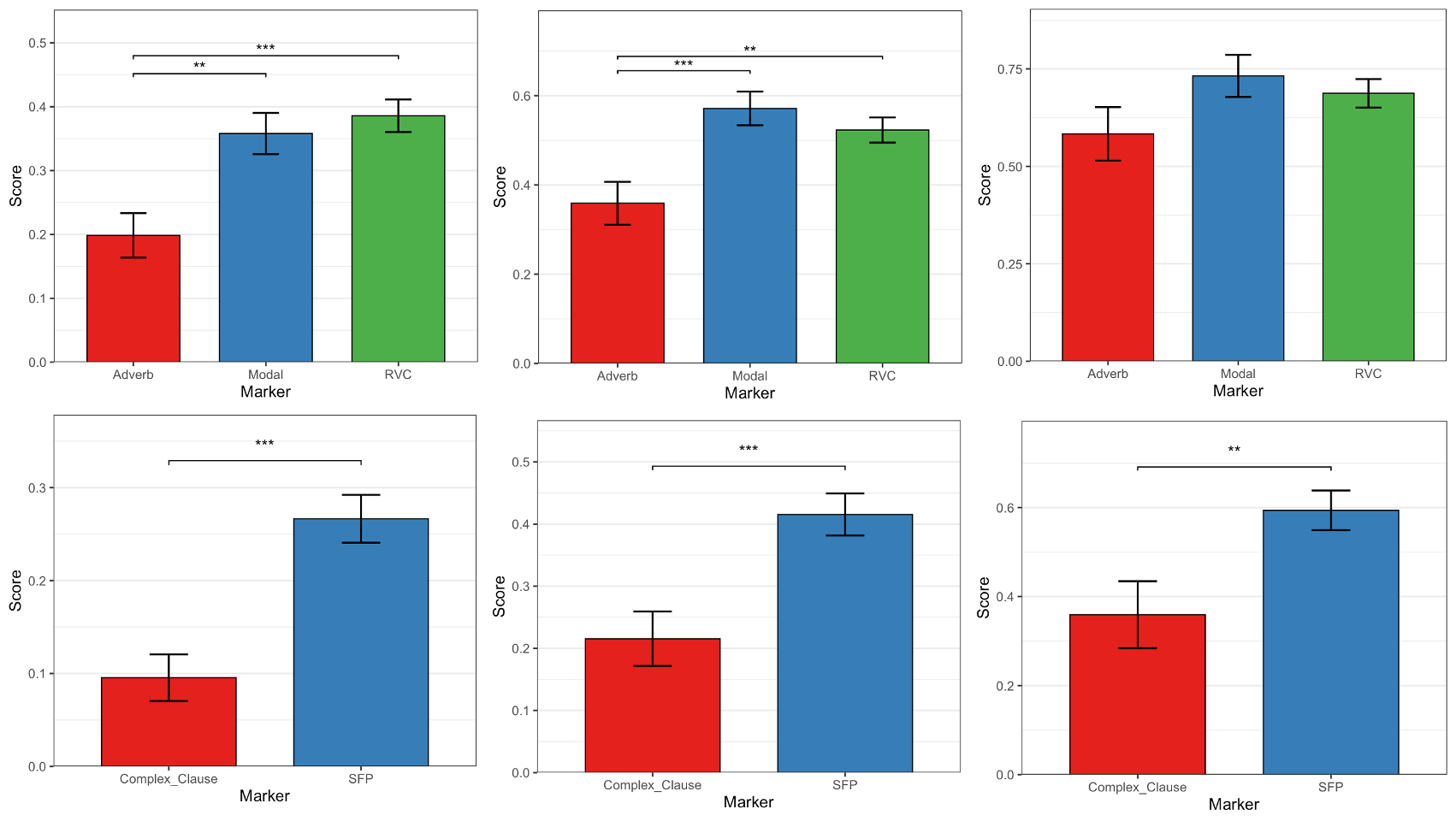


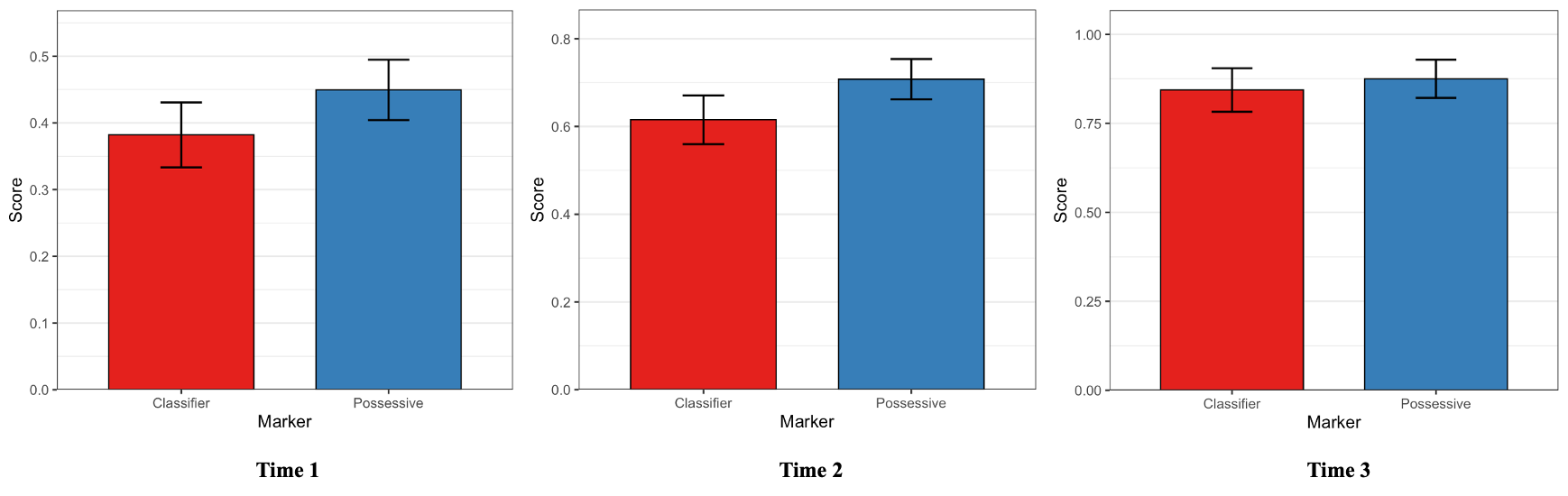


Supplementary Figure. Score comparison across grammatical categories and three time points
